# TLR4 Ligand/H_2_O_2_ Enhances TGF-β1 Signaling to Induce Metastatic Potential of Non-Invasive Breast Cancer Cells by Activating Non-Smad Pathways

**DOI:** 10.1371/journal.pone.0065906

**Published:** 2013-05-29

**Authors:** Yuan-Hong Zhou, Sheng-Jun Liao, Dong Li, Jing Luo, Jing-Jing Wei, Bin Yan, Rui Sun, Yu Shu, Qi Wang, Gui-Mei Zhang, Zuo-Hua Feng

**Affiliations:** Department of Biochemistry & Molecular Biology, Tongji Medical College, Huazhong University of Science & Technology, Wuhan, The People's Republic of China; Dartmouth, United States of America

## Abstract

TGF-β1 has the potential to activate multiple signaling pathways required for inducing metastatic potential of tumor cells. However, TGF-β1 was inefficient in inducing metastatic potential of many non-invasive human tumor cells. Here we report that the enhancement of TGF-β1 signaling is required for inducing metastatic potential of non-invasive breast cancer cells. TGF-β1 alone could not efficiently induce the sustained activation of Smad and non-Smad pathways in non-invasive breast cancer cells. TLR4 ligand (LPS) and H_2_O_2_ cooperated with TGF-β1 to enhance the sustained activation of non-Smad pathways, including p38MAPK, ERK, JNK, PI3K, and NF-κB. The activation of MAPK and PI3K pathways resulted in a positive feed-back effect on TGF-β1 signaling by down-regulating Nm23-H1 expression and up-regulating the expression of TβRI and TβRII, favoring further activation of multiple signaling pathways. Moreover, the enhanced TGF-β1 signaling induced higher expression of SNAI2, which also promoted TβRII expression. Therefore, the sustained activation levels of both Smad and non-Smad pathways were gradually increased after prolonged stimulation with TGF-β1/H_2_O_2_/LPS. Consistent with the activation pattern of signaling pathways, the invasive capacity and anoikis-resistance of non-invasive breast cancer cells were gradually increased after prolonged stimulation with TGF-β1/H_2_O_2_/LPS. The metastatic potential induced by TGF-β1/H_2_O_2_/LPS was sufficient for tumor cells to extravasate and form metastatic foci in an experimental metastasis model in nude mice. The findings in this study suggested that the enhanced signaling is required for inducing higher metastatic capacity of tumor cells, and that targeting one of stimuli or signaling pathways might be potential approach in comprehensive strategy for tumor therapy.

## Introduction

The efficient activation of multiple signaling pathways is the important driving force for tumor cell metastasis [1]–[3]. Compared with high-invasive human cancer cells, non-invasive human cancer cells have constitutively lower activation of signaling pathways [4], [5]. Given that tumor microenvironment can influence the metastatic capacity of tumor cells [6], the metastatic potential of non-invasive tumor cells might be induced by modulatory factor(s) in tumor milieu. Since the activation of single signaling pathway is not sufficient for inducing the metastasis of non-invasive tumor cells [4], [5], the factor(s) which could activate multiple signaling pathways might be responsible for inducing metastasis of non-invasive tumor cells.

Transforming growth factor β1 (TGF-β1), the most potent factor to induce epithelial to mesenchymal transition (EMT) [7], [8], has the potential to activate multiple signaling pathways, including Smad pathway and non-Smad pathways such as p38MAPK, ERK, JNK, PI3K, and NF-κB [8], [9]. The increased production of TGF-β1 has been observed in many types of carcinomas [7], [10]. The carcinomas with excess TGF-β1 production are more motile and invasive, and exhibit increased tumor cell metastasis in athymic mice [7]. All of these findings implicate the important roles of TGF-β1 in tumor metastasis. However, many non-invasive tumor cells could not undergo TGF-β1-induced EMT *in vitro* [7], suggesting that TGF-β1 might not be able to efficiently activate multiple signaling pathways in non-invasive tumor cells. The effect of TGF-β1 in tumor milieu might be enhanced by other factor(s) which could cooperate with TGF-β1 to induce sufficient activation of multiple signaling pathways, and promote the metastatic capacity, including invasion and extravasation, of tumor cells.

It has been found that TLR4 ligand and H_2_O_2_ also have the potential to activate non-Smad pathways which could be activated by TGF-β1 [11]–[16], suggesting the possibility that TLR4 ligand and/or H_2_O_2_ might cooperate with TGF-β1 to induce sufficient activation of multiple signaling pathways, favoring metastatic potential of non-invasive tumor cells. TLR4 ligands could be existent *in vivo* due to surgery, damage of tumor cells, or the existence of bacteria in tumor [17]–[20]. H_2_O_2_, one of the molecules involved in inflammation, is abundantly existent in tumor milieu [21]. Therefore, in this study we investigated whether TGF-β1 could induce the metastatic potential of non-invasive tumor cells in presence of TLR4 ligand and/or H_2_O_2_, and whether TLR4 ligand and/or H_2_O_2_ could enhance TGF-β1 signaling in non-invasive tumor cells. Our results showed that TLR4 ligand and H_2_O_2_ could cooperate with TGF-β1 to induce sustained activation of multiple signaling pathways in non-invasive human breast cancer cells, promoting the metastatic potential sufficient for invasion and extravasation of tumor cells.

## Results

### TGF-β1/H_2_O_2_/LPS promotes the invasive migration of non-invasive breast cancer cells

**Figure 1:**
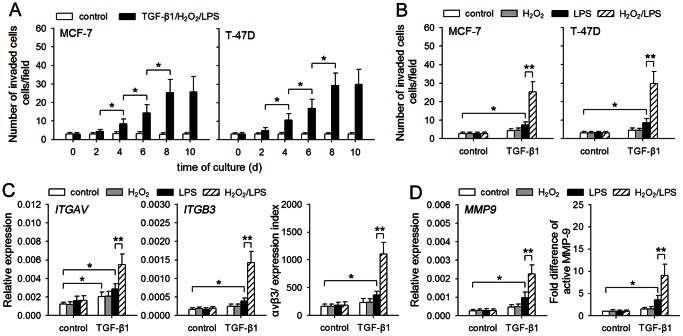
TGF-β1/H_2_O_2_/LPS facilitates invasive capability of non-invasive breast cancer cells. (**A**) MCF-7 and T-47D cells were cultured in absence or presence of TGF-β1 (5 ng/ml)/H_2_O_2_ (50 µM)/LPS (100 ng/ml) for the indicated time, and then used for Matrigel invasion assay. (**B–D**) Tumor cells were cultured in absence or presence of TGF-β1, H_2_O_2_ and LPS for 8 days. The cells were then used for following experiments. (**B**) The cells were used for Matrigel invasion assay. (**C**) The expression of αvβ3 was analyzed by real-time RT-PCR and flow cytometry. αvβ3 expression index was calculated as described in Materials and Methods. (**D**) The cells were then cultured in presence of matrigel for 48 h. The mRNA level of *MMP9* was detected by real-time RT-PCR. The MMP-9 in supernatants was detected by zymography assay, and the fold difference of active MMP-9 was calculated after densitometric analysis of the gel. *P* values, *P<0.05, **P<0.01.

To investigate whether TGF-β1, H_2_O_2_, and TLR4 ligand might cooperate to promote invasive migration of non-invasive breast cancer cells, we first cultured non-invasive MCF-7 and T-47D cells in presence of TGF-β1, H_2_O_2_, and LPS (a well known TLR4 ligand). The capacity of invasive migration of tumor cells was gradually increased after prolonged stimulation (Figure 1A). We then further analyzed the effects of TGF-β1, H_2_O_2_, and LPS. The result showed that H_2_O_2_ and LPS, alone or in combination, could not influence the invasive migration of these cells (Figure 1B). TGF-β1, alone or in combination with either H_2_O_2_ or LPS, slightly promoted the invasive migration of MCF-7 and T-47D cells. However, the invasive migration of these cells was much more efficient after treatment with TGF-β1/H_2_O_2_/LPS (Figure 1B). Consistently, the polymerization of actin in tumor cells in response to ECM molecules (matrigel), which is important for migratory and invasive properties of tumor cells [22], was also increased by TGF-β1/H_2_O_2_/LPS (Figure S1). Moreover, TGF-β1/H_2_O_2_/LPS significantly up-regulated the expression of αvβ3 (Figure 1C), which is the key integrin mediating tumor cell arrest during flow and the invasive migration of tumor cells [4]. The production of active MMP-9 in response to ECM was also increased by pretreatment with TGF-β1/H_2_O_2_/LPS (Figure 1D). Taken together, these results indicated that TGF-β1, H_2_O_2_, and LPS could cooperate to promote the invasive migration of non-invasive breast cancer cells.

### Sustained activation of non-Smad pathways is enhanced by co-stimulation with TGF-β1/H_2_O_2_/LPS

**Figure 2:**
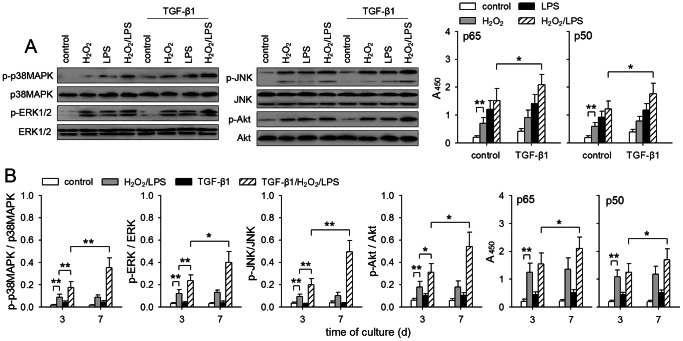
Co-stimulation with TGF-β1/H_2_O_2_/LPS enhances the sustained activation of non-Smad pathways. (**A**) MCF-7 cells were cultured for 7 days in absence or presence of TGF-β1 (5 ng/ml), H_2_O_2_ (50 µM), and LPS (100 ng/ml). The phosphorylated and un-phosphorylated p38MAPK, ERK, JNK, and Akt were detected by Western blot. The activity of NF-κB was assayed as described in Methods. (**B**) MCF-7 cells were cultured for the indicated time in absence or presence of TGF-β1 and/or H_2_O_2_/LPS. The ratio of phosphorylated and un-phosphorylated p38MAPK, ERK, JNK, and Akt was calculated after densitometric analysis of Western blots. The activity of NF-κB was assayed as described in Methods. *P* values, *P<0.05, **P<0.01.

The requirement for prolonged stimulation implied that the sustained activation of signaling pathways was important for TGF-β1/H_2_O_2_/LPS to promote invasive migration of non-invasive breast cancer cells. We then analyzed the effect of TGF-β1/H_2_O_2_/LPS on the sustained activation of non-Smad pathways. To do this, we first detected the sustained activation of these pathways by stimulating MCF-7 cells for 7 days with TGF-β1, H_2_O_2_, and LPS. The result showed that the co-stimulation with three stimuli was most efficient in activating non-Smad pathways (Figure 2A), suggesting that the cooperation of all three stimuli was required for the most efficient activation of these pathways. Intriguingly, the prolonged stimulation resulted in the enhanced activation of these pathways (Figure 2B), suggesting that the signaling was amplified after prolonged stimulation.

### Sustained activation of Smad pathway is enhanced by co-stimulation with TGF-β1/H_2_O_2_/LPS

**Figure 3:**
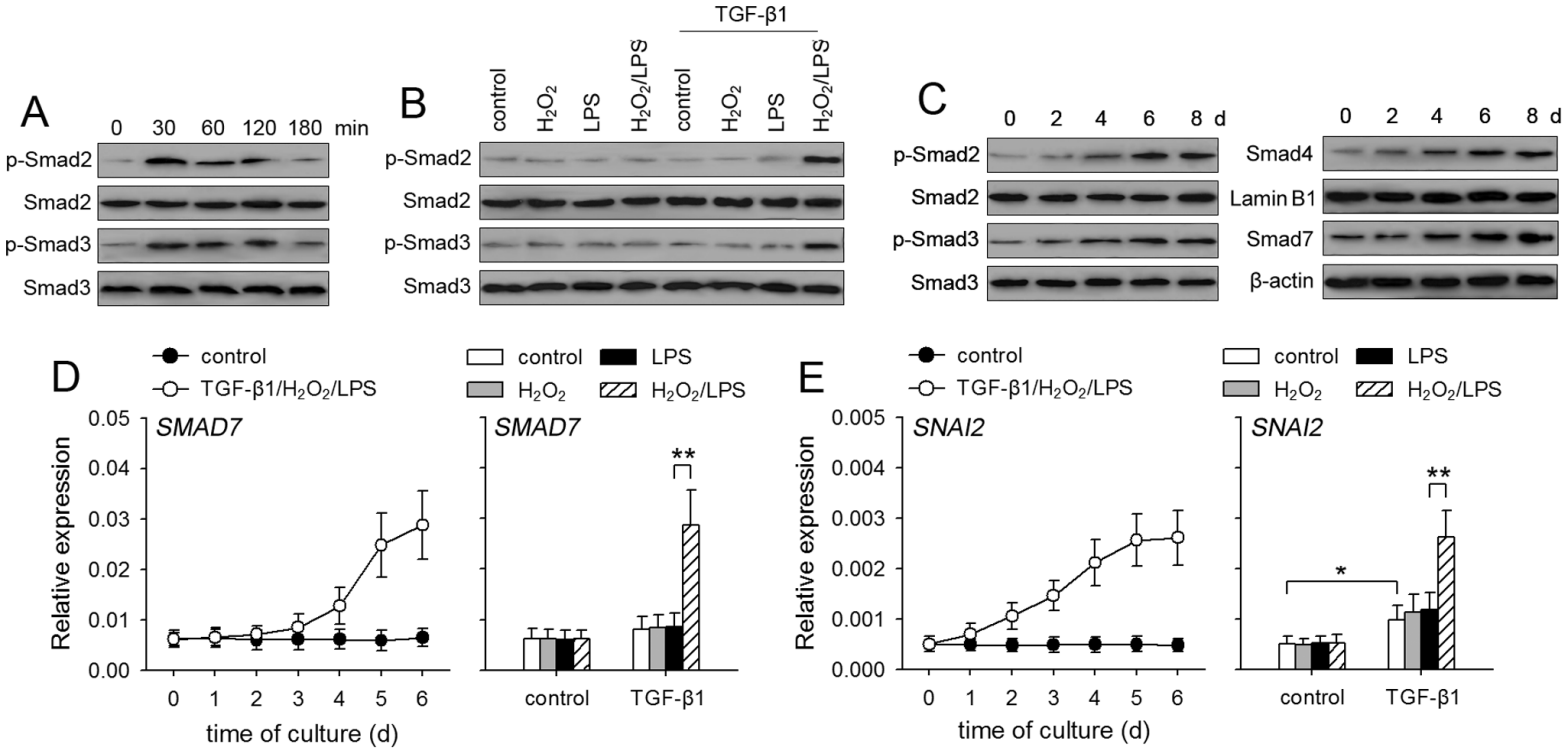
H_2_O_2_ and LPS enhance the sustained activation of Smad pathway. (**A**) MCF-7 cells were stimulated with TGF-β1 (5 ng/ml). Phospho-Smad2, Smad2, phospho-Smad3 and Smad3 were detected by Western blot at the indicated time points. (**B**) MCF-7 cells were cultured in absence or presence of TGF-β1, H_2_O_2_ (50 µM), and LPS (100 ng/ml) for 7 days. Phospho-Smad2, Smad2, phospho-Smad3 and Smad3 were detected by Western blot. (**C**) MCF-7 cells were cultured in presence of TGF-β1/H_2_O_2_/LPS. Phospho-Smad2, Smad2, phospho-Smad3, Smad3, Smad7, and β-actin were detected by Western blot at the indicated time points. Smad4 in nuclear extract was detected by Western blot. Lamin B1, a nuclear protein, in nuclear extract was used as control. (**D**) MCF-7 cells were cultured in absence or presence of TGF-β1/H_2_O_2_/LPS (left) for the indicated time. Or the cells were cultured for 6 days in absence or presence of TGF-β1, H_2_O_2_, and LPS (right). The expression of *SMAD7* (**D**) and *SNAI2* (**E**) were detected by real-time RT-PCR. *P* values, *P<0.05, **P<0.01.

The above data showed that TGF-β1 alone was inefficient in inducing the sustained activation of non-Smad pathways. We then asked whether the sustained activation of Smad pathway in non-invasive breast cancer cells could be induced by TGF-β1. TGF-β1 alone induced transient activation of Smad2/3 in MCF-7 cells (Figure 3A), but could not induce the sustained activation of Smad2/3, evaluated by the levels of p-Smad2 and p-Smad3 after 7-d stimulation (Figure 3B). Intriguingly, TGF-β1 could induce the sustained activation of Smad2/3 in presence of both H_2_O_2_ and LPS (Figure 3B). The sustained activation of Smad2/3 in MCF-7 cells was gradually enhanced in presence of TGF-β1/H_2_O_2_/LPS (Figure 3C). In accordance with the gradually enhanced activation of Smad2/3, the nuclear translocation of Smad4 was gradually increased by TGF-β1/H_2_O_2_/LPS (Figure 3C). The expression of Smad7, which is induced by TGF-β1-Smad pathway [23], was gradually up-regulated by TGF-β1/H_2_O_2_/LPS (Figure 3C, 3D). In addition to Smad7, TGF-β1/H_2_O_2_/LPS also increased the expression of SNAI2 (Figure 3E), a member of Snail family mediating the effect of TGF-β1, which could be up-regulated by Smad and ERK pathways [8], [24]. These data suggested that H_2_O_2_ and LPS enhanced TGF-β1-induced sustained activation of not only non-Smad pathways but also Smad pathway. However, although Smad pathway could modulate the expression of p21 and MYC [25], the expression of p21 and MYC was not significantly influenced by TGF-β1/H_2_O_2_/LPS (Figure S2), possibly due to the opposite effects of Smad pathway and non-Smad pathways on the expression of p21 and MYC [25]–[28].

### TGF-β1 signaling is enhanced by up-regulating TGF-β receptors and down-regulating Nm23-H1

**Figure 4:**
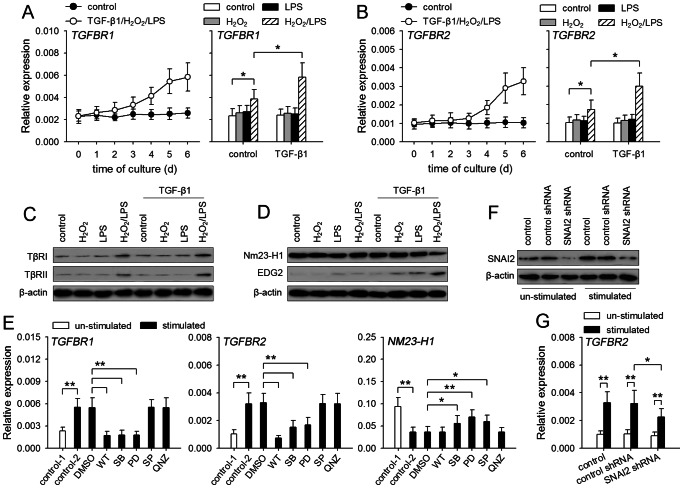
TGF-β1/H_2_O_2_/LPS down-regulates Nm23-H1 expression and up-regulates the expression of TGF-β receptors. (**A–C**) MCF-7 cells were cultured in absence or presence of TGF-β1/H_2_O_2_/LPS (left) for the indicated time. Or the cells were cultured for 6 days in absence or presence of TGF-β1, H_2_O_2_, and LPS (right). The expression of *TGFBR1* (**A**), and *TGFBR2* (**B**) was detected by real-time RT-PCR. The expression of TβRI and TβRII was detected by Western blot after 6-d culture (**C**). (**D**) MCF-7 cells were cultured for 6 days in absence or presence of TGF-β1, H_2_O_2_, and LPS. The expression of Nm23-H1 and EDG2 was detected by Western blot. (**E**) MCF-7 cells were un-stimulated or stimulated for 6 days with TGF-β1/H_2_O_2_/LPS in absence or presence of wortmannin (WT, 40 nM), SB203580 (SB, 10 µM), PD98059 (PD, 10 µM), SP600125 (SP, 10 µM) or QNZ (40 nM). The expression of *TGFBR1*, *TGFBR2* and *NM23-H1* was detected by real-time RT-PCR. (**F and G**) Control MCF-7 cells and the MCF-7 cells expressing control shRNA or SNAI2 shRNA were untreated or treated with TGF-β1/H_2_O_2_/LPS for 6 days. SNAI2 expression was detected by Western blot (**F**). The expression of *TGFBR2* was detected by real-time RT-PCR (**G**). *P* values, *P<0.05, **P<0.01.

To further investigate the mechanisms underlying the enhanced activation of Smad and non-Smad pathways, we analyzed whether the expression of TLR4 and TGF-β receptors was influenced by TGF-β1/H_2_O_2_/LPS. The expression of TLR4 was not significantly changed after stimulation with three stimuli (data not shown). In presence of TGF-β1/H_2_O_2_/LPS, the expression of TβRI and TβRII was gradually increased after prolonged stimulation (Figure 4A, 4B). TGF-β1, H_2_O_2_ or LPS alone could not influence TβRI and TβRII expression. H_2_O_2_ and LPS could cooperate to promote the expression of these receptors, and cooperate with TGF-β1 to further up-regulate the expression of these receptors (Figure 4A, 4B, 4C).

Since the expression of Smad7, the feed-back inhibitor of Smad2/3 activation, was not suppressed (Figure 3D), we next investigated whether TGF-β1/H_2_O_2_/LPS might modulate the expression of Nm23-H1 which has been found to negatively regulate TGF-β signaling [29]. The result showed that the expression of Nm23-H1 in the cells was significantly reduced by TGF-β1/H_2_O_2_/LPS, but not one or two of them (Figure S3, 4D). The down-regulation of Nm23-H1 was further proved by the increased expression of EDG2 (Figure S3, 4D), since Nm23-H1 has been known to suppress EDG2 expression [30].

We then analyzed whether non-Smad pathways were involved in modulating the expression of Nm23-H1 and TGF-β receptors, since TLR4 ligand and H_2_O_2_ were required for the modulation. To do this, we detected the mRNA levels of TβRI, TβRII, and Nm23-H1 after stimulation with TGF-β1/H_2_O_2_/LPS in presence of wortmannin (PI3K inhibitor), SB203580 (p38MAPK inhibitor), PD98059 (inhibitor of ERK pathway), SP600125 (JNK inhibitor), QNZ (NF-κB inhibitor). The results showed that PI3K, p38MAPK and ERK pathways were required for up-regulating the expression of TβRI and TβRII, and that p38MAPK, ERK and JNK pathways were involved in down-regulating Nm23-H1 expression (Figure 4E). As shown in Figure 3E, TGF-β1/H_2_O_2_/LPS could up-regulate the expression of SNAI2, which also promotes the expression of TβRII [31]. We then used SNAI2 shRNA to inhibit TGF-β1/H_2_O_2_/LPS-induced expression of SNAI2 (Figure 4F), which partially, but significantly, hindered the up-regulation of TβRII by TGF-β1/H_2_O_2_/LPS (Figure 4G).

To further clarify the effect of the increased expression of TβRI and TβRII on the sustained activation of signaling pathways, we used PI3K inhibitor to suppress the up-regulation of TβRI and TβRII. PI3K inhibitor not only suppressed the up-regulation of TβRI and TβRII, but also hindered the increase of the activation levels of other non-Smad pathways (Figure S4), suggesting that the increased expression of TGF-β receptors was important for inducing higher activation levels of signaling pathways.

### Anoikis-resistance of non-invasive breast cancer cells is promoted by TGF-β1/H_2_O_2_/LPS

**Figure 5:**
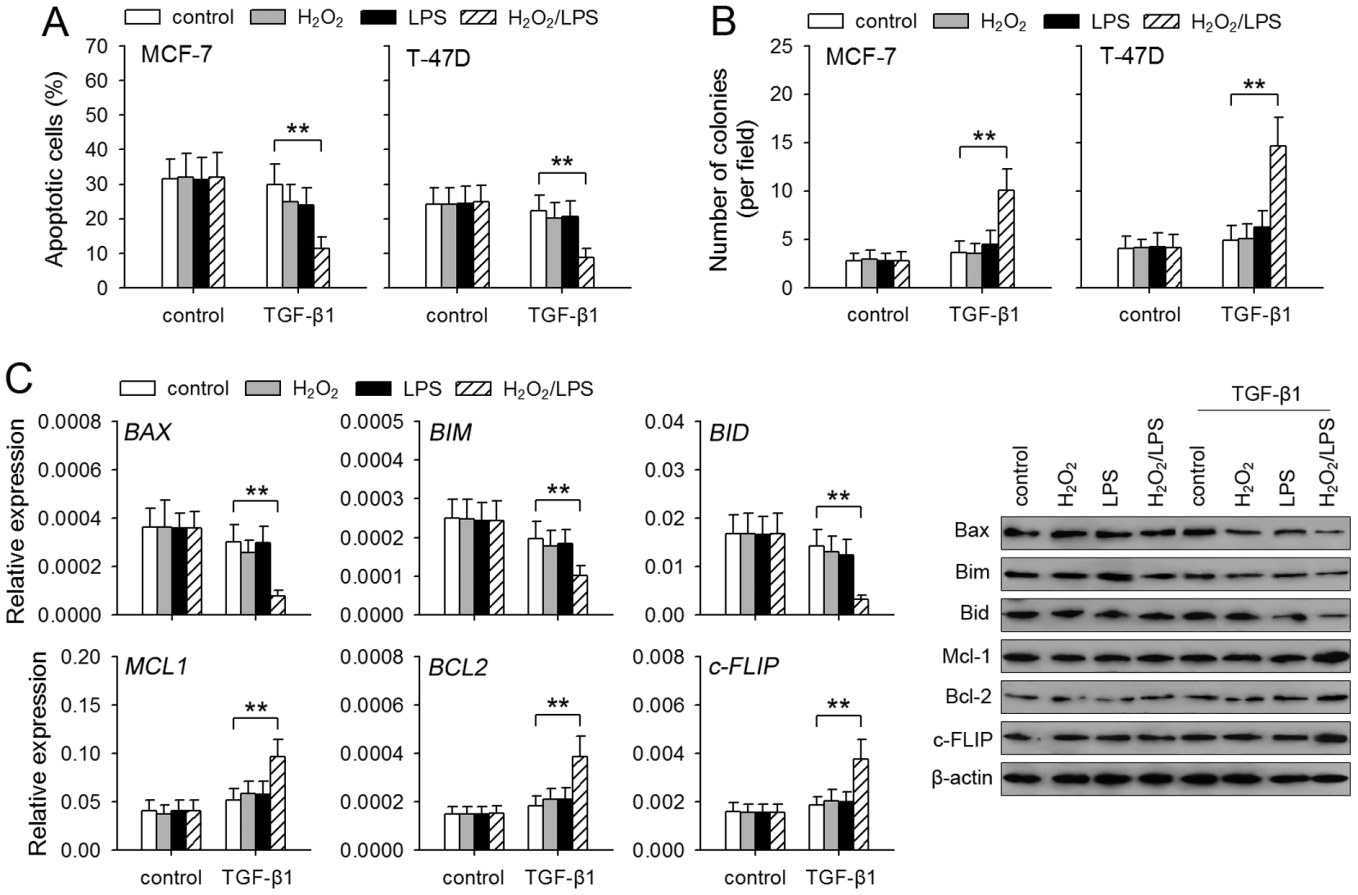
TGF-β1/H_2_O_2_/LPS promotes anoikis-resistance of non-invasive breast cancer cells. MCF-7 and T-47D cells were cultured for 8 days in absence or presence of TGF-β1 (5 ng/ml), H_2_O_2_ (50 µM), and LPS (100 ng/ml). The cells were then used in following experiments. (**A**) The cells were transferred to poly-HEMA-coated plate and cultured for 24 h. The apoptosis of the cells was analyzed by flow cytometry. (**B**) The cells were then cultured in soft agar for 3 weeks. The colonies were counted. (**C**) MCF-7 cells were used for the assay of the expression of Bax, Bim, Bid, Bcl-2, Mcl-1 and c-FLIP. The expression of these genes was detected by real-time RT-PCR and Western blot. *P* values, *P<0.05, **P<0.01.

In addition to invasive migration, anoikis-resistance is also required for metastasis of tumor cells [32], [33]. We next investigated whether TGF-β1/H_2_O_2_/LPS could influence the anoikis-resistance of MCF-7 and T-47D cells. To test this, we detected the apoptosis of tumor cells by culturing the cells under anchorage-independent condition after pre-treatment with TGF-β1, H_2_O_2_, and LPS. The result showed that TGF-β1/H_2_O_2_/LPS gradually increased the anoikis-resistance of MCF-7 and T-47D cells (Figure S5). Pre-treatment with TGF-β1/H_2_O_2_/LPS significantly promoted the anoikis-resistance of the cells, whereas none of them alone could significantly influence the anoikis-resistance (Figure 5A). Moreover, TGF-β1/H_2_O_2_/LPS-treated cells formed more colonies after culture in soft-agar (Figure 5B), further indicating that the anoikis-resistance of tumor cells was increased.

Both mitochondrial and extrinsic pathways are involved in anoikis [32], [33]. We then further analyzed the expression of several representative genes influencing mitochondrial pathway (Bax, Bim, Bid, Bcl-2 and Mcl-1) and extrinsic pathway (c-FLIP). The results showed that the co-stimulation with TGF-β1/H_2_O_2_/LPS, but not each of them alone, up-regulated the expression of anti-apoptotic genes (Bcl-2, Mcl-1, c-FLIP), and down-regulated the expression of pro-apoptotic genes (Bax, Bim, Bid) (Figure 5C).

### TGF-β1/H_2_O_2_/LPS promotes the capacity of tumor cells to extravasate and form metastatic foci

**Figure 6:**
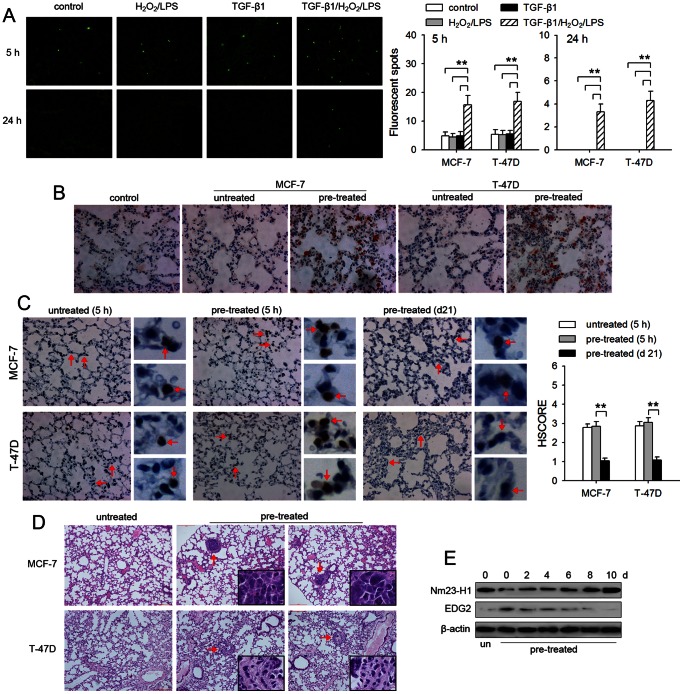
TGF-β1/H_2_O_2_/LPS promotes the capacity of tumor cells to extravasate and form metastatic foci. (**A**) MCF-7 and T-47D cells were cultured for 8 days in absence or presence of TGF-β1 (5 ng/ml) and/or H_2_O_2_ (50 µM)/LPS (100 ng/ml). The cells were then labeled with CFSE and injected to mice via tail vein. Mice were sacrificed 5 h and 24 h after injection. The CFSE-labeled tumor cells in frozen sections were visualized by fluorescence microscopy (left). Fluorescent spots in the frozen sections of lung tissues were counted (right). (**B–D**) MCF-7 and T-47D cells were untreated or pre-treated for 8 days with TGF-β1/H_2_O_2_/LPS. The cells were then injected to mice via tail vein. The mice without inoculation with tumor cells were used as control. Mice (n = 6 per group) were sacrificed on d21 after inoculation (**B**) or at the indicated time after inoculation **(C)**. The sections of lung tissues were prepared and subjected to immunohistochemical analysis. The tissue sections were stained with anti-human MGB1 antibody (**B**) or anti-human Ki-67 antibody (**C**). In each photograph in **C**, 2 representative sites (indicated by arrows) were further amplified to show the expression of Ki-67 in tumor cells (indicated by arrows). The immunohistochemical staining intensity of Ki-67 was measured using the HSCORE scoring system (right) as described in Methods. (**D**) The mice (n = 6 per group) were sacrificed 8 weeks after inoculation. The sections of lung tissues were prepared and subjected to H&E staining. Insets are the high-power view of metastatic foci in corresponding picture indicated by arrows. Representative photographs are shown (**B–D**). (**E**) MCF-7 and T-47D cells were untreated (un) or pre-treated for 8 days with TGF-β1/H_2_O_2_/LPS. The cells were then cultured in absence of stimuli for indicated time. The expression of Nm23-H1 and EDG2 was detected by Western blot. *P* values, **P<0.01.

We next tested the metastatic potential of tumor cells in an experimental metastasis model in nude mice. CFSE-labeled MCF-7 and T-47D cells were injected into nude mice via tail vein. Tumor cell arrest in lung was significantly increased in TGF-β1/H_2_O_2_/LPS-treatment group, evaluated by the fluorescent spots in lung tissues 5 h after i.v. injection (Figure 6A). 24 h later, fluorescent spots were only observed in TGF-β1/H_2_O_2_/LPS-treatment group, but not in other groups (Figure 6A), suggesting that TGF-β1/H_2_O_2_/LPS-induced metastatic potential was sufficient for tumor cells to extravasate into lung tissue from circulation.

To confirm the capacity of disseminated tumor cells to form metastatic lesions after extravasation, we analyzed lung tissue by immunohistochemical staining 3 weeks after inoculation. Tumor cells were observed in the lung tissue of each mouse in TGF-β1/H_2_O_2_/LPS-treatment group (6/6), but not in other groups (P = 0.001, Fisher's Exact Test), evaluated by positive staining for a marker (MGB1) of breast cancer cells (Figure 6B). In TGF-β1/H_2_O_2_/LPS-treatment group, the number of tumor cells in lung tissue was increased (Figure 6B), compared with that in lung tissue 24 h after inoculation as shown in Figure 6A. Nevertheless, the accumulation of Ki-67, a proliferation marker, in nucleus was significantly lower in the tumor cells in lung tissue 3 weeks after inoculation, compared with that in the tumor cells 5 h after inoculation (Figure 6C). The reduced expression of Ki-67 suggested that the disseminated tumor cells slowly proliferated in metastatic sites. Consistently, metastatic foci (Figure 6D), but not visible metastatic nodules, were observed in lung 8 weeks after inoculation. The metastatic foci were existent in the lung tissues of 5/6 of mice (MCF-7) or 6/6 of mice (T-47D) in TGF-β1/H_2_O_2_/LPS-treatment groups, but not in other groups (MCF-7, P = 0.008; T-47D, P = 0.001; Fisher's Exact Test).

TGF-β1/H_2_O_2_/LPS reduced the expression of Nm23-H1, and therefore increased the expression of EDG2, which should be in favor of the proliferation of disseminated tumor cells [34], [35]. To elucidate the slower proliferation of tumor cells after extravasation, we analyzed the expression of Nm23-H1 and EDG2 in the tumor cells after withdrawal of stimuli. The result showed that the expression of Nm23-H1 was gradually increased, whereas EDG2 expression was gradually reduced after withdrawal of TGF-β1/H_2_O_2_/LPS (Figure 6E), suggesting that the reversed expression of these genes might, at least in part, restrict the development of metastatic lesions.

## Discussion

Although TGF-β1 has the potential to activate multiple signaling pathways, our data in this study showed that TGF-β1 alone was inefficient in inducing the sustained activation of Smad and non-Smad pathways in non-invasive breast cancer cells, which could explain the inefficiency of TGF-β1 in inducing EMT of non-invasive tumor cells *in vitro* reported previously [7]. Importantly, in this study we demonstrated that TGF-β1 signaling was significantly enhanced by H_2_O_2_ and TLR4 ligand (LPS), thus promoting the invasive migration and anoikis-resistance of non-invasive breast cancer cells. The enhanced TGF-β1 signaling by TLR4 ligand and H_2_O_2_ might explain the effect of TGF-β1 *in vivo*.

When non-invasive MCF-7 and T-47D breast cancer cells were stimulated with TGF-β1, the sustained activation of non-Smad pathways was limited. Both H_2_O_2_ and LPS could activate non-Smad pathways, including p38MAPK, ERK, JNK, PI3K, and NF-κB. Co-stimulation with H_2_O_2_ and LPS could enhance the activation of these pathways to some extent in non-invasive breast cancer cells. Moreover, the stimulation with TGF-β1/H_2_O_2_/LPS could further promote the sustained activation of these pathways, indicating that the co-stimulation was more efficient in activating non-Smad pathways in non-invasive breast cancer cells.

In addition to co-stimulating, the feed-back regulation was also involved in inducing the sustained and higher activation levels of non-Smad pathways. The enhanced activation of PI3K and MAPK pathways by co-stimulation resulted in a positive feed-back effect on the sustained activation of non-Smad pathways. By enhancing the activation of PI3K and MAPK pathways, H_2_O_2_/LPS could directly promote TβRI and TβRII expression, and cooperate with TGF-β1 to further up-regulate the expression of these receptors. The increased receptors could amplify the activating effect of TGF-β1 on non-Smad pathways. Therefore, TGF-β1/H_2_O_2_/LPS-induced activation of non-Smad pathways could be gradually enhanced after prolonged stimulation. The increased expression of TGF-β receptors was important for the gradual increase of the activation levels of non-Smad pathways, since inhibiting the up-regulation of TGF-β receptors with PI3K inhibitor could hinder the further activation of all other non-Smad pathways.

H_2_O_2_/LPS could not directly activate Smad pathway. However, the sustained activation of PI3K and MAPK pathways could enhance TGF-β1-induced activation of Smad pathway by up-regulating TβRI expression and down-regulating Nm23-H1 expression. Smad7 is induced by TGF-β-Smad pathway, and in turn, inhibits Smad2/3 activation by competitively binding to TβRI and inducing the degradation of TGF-β receptors [7], [9], [23]. H_2_O_2_ and LPS could cooperate to promote TβRI expression, and cooperate with TGF-β1 to further up-regulate the expression of TβRI by activating PI3K and MAPK pathways. The up-regulation of TβRI might, at least in part, counteract the inhibitory effect of Smad7. On the other hand, Nm23-H1 negatively regulates the activation of Smad2/3 by promoting the recruitment of Smad7 to TβRI and stabilizing the binding of Smad7 with TβRI [29]. The enhanced activation of non-Smad pathways resulted in the down-regulation of Nm23-H1 expression. Since Nm23-H1 negatively regulates TGF-β1 signaling in a dose-dependent manner [29], the down-regulation of Nm23-H1 by TGF-β1/H_2_O_2_/LPS might result in the attenuation of the interaction between Smad7 and TβRI, favoring the activation of Smad2/3. Therefore, as shown by our data, TGF-β1 could induce the sustained activation of Smad2/3 in non-invasive breast cancer cells in presence of H_2_O_2_ and LPS.

The activation of Smad and ERK pathways is required for up-regulating the expression of SNAI2 [8], [24]. The increased expression of SNAI2 was a part of positive feed-back loop which enhanced TGF-β1 signaling, since SNAI2 promoted the expression of TβRII as shown by our data and others [31]. Therefore, both Smad and non-Smad pathways were involved in the feed-back regulation of TGF-β1 signaling. Nevertheless, the feed-back effect was only induced by TGF-β1 in presence of H_2_O_2_ and LPS, but not by TGF-β1 alone, suggesting that TGF-β1 alone could not switch on the positive feed-back loop in non-invasive tumor cells. The enhanced activation of non-Smad pathways by co-stimulation with TGF-β1/H_2_O_2_/LPS was sufficient to induce positive feed-back effect, thus gradually enhancing the activation of Smad and non-Smad pathways. Therefore, the activation of Smad and non-Smad pathways induced by TGF-β1 in presence of H_2_O_2_ and LPS was sufficient to modulate the metastatic potential of non-invasive breast cancer cells.

TGF-β1 could induce apoptosis in a Smad-dependent manner in some types of tumor cells [36]. However, the sustained activation of MAPK pathways could promote apoptosis-resistance of tumor cells, and abolish TGF-β1-indced apoptosis [36]. In addition, the activation of NF-κB leads to the transcriptional activation of genes that suppress apoptosis [37]. H_2_O_2_/LPS-enhanced TGF-β1 signaling not only induced the sustained activation of Smad pathway, but also induced the sustained and higher activation levels of non-Smad pathways. Therefore, the enhanced TGF-β1 signaling did not promote apoptosis in non-invasive breast cancer cells. In contrast, TGF-β1/H_2_O_2_/LPS promoted not only the invasive migration but also the anoikis-resistance of breast cancer cells.

TGF-β1/H_2_O_2_/LPS-induced metastatic potential was sufficient for tumor cell extravasation as shown by our data in animal test. The disseminated tumor cells slowly developed into metastatic foci due to lower proliferation at metastatic sites, since the tumor cells at metastatic sites showed lower expression of proliferation marker Ki-67. It has been known that new microenvironment could restrict the proliferation of disseminated tumor cells [38]. Different from high-invasive tumor cells, the metastatic tumor cells derived from non-invasive tumor cells might be more sensitive to the restriction of new microenvironment. On the other hand, it has been found that Nm23-H1 has the potential to hinder the growth of disseminated tumor cells at the metastatic site [34], and that EDG2 is required for the proliferation of disseminated tumor cells in new microenvironment [35]. TGF-β1/H_2_O_2_/LPS-modulated expression of Nm23-H1 and EDG2 was reversed when tumor cells were away from the stimuli, which might partially explain the slower development of metastatic lesions.

In summary, in this study we demonstrated that H_2_O_2_/LPS could enhance TGF-β1 signaling to induce the sustained activation of both Smad and non-Smad pathways in non-invasive breast cancer cells, and thus promoting the metastatic capability of non-invasive breast cancer cells. Metastases are responsible for most cancer deaths. Given that the enhanced signaling is required for inducing higher metastatic capacity of tumor cells, targeting one of these stimuli or signaling pathways might be potential approach in comprehensive strategy for tumor therapy.

## Materials and Methods

### Ethics statement

All animal works were conducted according to relevant national and international guidelines. They were approved by the Committee on the Ethics of Animal Experiments of Tongji Medical College (Permit Number: 2010-S260) and monitored by the Department of Experimental Animals of Tongji Medical College.

### Cells and reagents

MCF-7 and T-47D cell lines were purchased from the Type Culture Collection of the Chinese Academy of Sciences (Shanghai, China), and cultured according to their guidelines. TGF-β1 was purchased from PeproTech (Rocky Hill, NJ). H_2_O_2_ (hydrogen peroxide) and LPS (lipopolysaccharide) were purchased from Sigma-Aldrich (St. Louis, MO). Matrigel was purchased from BD Biosciences (Bedford). SB203580, wortmannin, PD98059, SP600125, 6-amino-4-(4-phenoxyphenylethylamino) quinazoline (QNZ) were purchased from Merck4 Biosciences (Calbiochem).

### Matrigel invasion assay

Matrigel invasion assay was performed using Boyden chambers (Transwell, Corning, Inc., Corning, NY). The transwell filter inserts were coated with matrigel. The lower chambers were filled with DMEM medium containing 10% FBS. 1×10^5^ tumor cells were placed in the upper compartment. After 24-h incubation at 37°C in a humidified incubator with 5% CO_2_, the non-invading cells were removed. The invasive cells attached to the lower surface of membrane insert were fixed, stained, and counted under a microscope from 7 randomly chosen fields in each membrane. The average number of the cells per field was calculated.

### Analysis for actin polymerization

Tumor cells were incubated in matrigel-coated plate for 5 h. The cells were then fixed in 4% paraformaldehyde, permeabilized with 0.1% Triton X-100, and then stained with rhodamine-phalloidin (Invitrogen) according to the manufacturer's protocol to visualize the cells with highly polymerized actin.

### Assay of gene expression by real-time RT-PCR

Total RNA was extracted from cells with TRIzol reagent (Invitrogen). The relative quantity of mRNA was determined by real-time RT-PCR according to MIQE guidelines [39]. Quantification of the expression of genes was performed using the comparative C_T_ method. *GAPDH*, *HPRT1* and *YWHAZ* were selected as reference genes according to the rules described in MIQE guidelines. The relative expression of the genes of interest was expressed as the mRNA level of the gene relative to the geometric mean of the mRNA levels of three reference genes, which was calculated using GeNorm software. The primer sequences were as follows: *NM23-H1*, sense 5′-TCATTGCGATCAAACCAGAT- 3′, antisense 5′-CAACGTAGT GTTC CTTGAGA-3′; *EDG2*, sense 5′-GCTATGTTCGCCAGAGGACT-3′, antisense 5′-ATC CAGGAGTCCAGCAGATG-3′; *ITGAV*, sense 5′-CTCGGGACTCCTGCTACCTC-3′, anti-sense 5′- AAGAAACATCCGGGAAGACG-3′; *ITGB3*, sense 5′-CATCCTGGTGGTCCT GCTCT-3′, antisense 5′-GCCTCTTTACACAGTGGGTTGTT-3′; *MMP9*, sense 5′-CAGTCCA CCCTTGTGCTCTTCC-3′, antisense 5′-CTGCCACCCGAGTGTAACCAT-3′; *BAX*, sense 5′-TTTTGCTTCAGGGTTTCATC-3′, antisense 5′-GACACTCGCTCAGCTTCTTG-3′; *BIM*, sense 5′-CA GAGCCACAAGACAGGA-3′, antisense 5′-CCATACAAATCTAAGCCAGT-3′; *BID*, sense 5′-GCCGTCCTTGCTCCGTGAT-3′, antisense 5′-ATGCCAGGGCTCCGTCTA-3′; *BCL2*, sense 5′-GGTCATGTGTGTGGAGAGC-3′, antisense 5′-GATCCAGGTGTGCAGGT G-3′; *MCL1*, sense 5′-TTGACTTCTGTTTGTCTTA CGCT-3′, antisense 5′-TGGTCCTAACC CTTCCTGG-3′; *c-FLIP*, sense 5′-AGAGTGAGGCGATTTGACCTG-3′, antisense 5′-AAG GTGAGGGTTCCTGAGCA-3′; *TGFBR1*, sense 5′-TGAACAGAAGTTAAGGCCAAATAT C-3′, antisense 5′-CAGGCAAAGCTGTAGAATTACATTT-3′; *TGFBR2*, sense 5′-CGGTTAA TAACGACATGATAGTCAC-3′, anti-sense 5′-TCATGGCAAACTGTCTCTAGTGTTA-3′; *SNAI2*, sense 5′-AGGAATCTGGCTGCTGTG-3′, antisense 5′-GGAGAAAATGCCTTTGGA C-3′; *SMAD7*, sense 5′-TCCTCCTGAGTGCTTGCTT-3′, antisense 5′-TCTGCTTCCCCTCT TCCTA-3′; *p21*, sense 5′-GGACAGCAGAGGAAGACCATGT-3′, antisense 5′-TGGAGTG GTAGAAATCTGTCATGC-3′; *MYC*, sense 5′-GCCACGTCTCCACACATCAG-3′, antisense 5′-TGGTGCATTTTCGGTTGTTG-3′; *GAPDH*, sense 5′-TCATTGACCTCAACT ACATGGTTT-3′, antisense 5′-GAAGATGGTGATGGGATTTC-3′; *HPRT1*, sense 5′-GCTG AGGATTTGGAA AGGGTG-3′, antisense 5′-CAGAGGGCTACAATGTGATGG-3′; *YWHAZ*, sense 5′-GATCTTTCTGGCTCCACTCA-3′, antisense 5′-CCATTCAGGATAGGTAGGGT-3′.

### Western blot assay

Western blot assay was done as described previously [4]. Abs were purchased from Santa Cruz Biotechnology and Cell Signaling Technology.

### Flow cytometric analysis

Tumor cells were stained with FITC-conjugated mouse-anti-human αvβ3 (Chemicon) or isotype control IgG1 for flow cytometric analysis. Parameters were acquired on a FACS Calibur 440E flow cytometer (BD Biosciences) and analyzed with CellQuest software (BD Biosciences). Percent staining was defined as the percentage of cells in the gate (M1) which was set to exclude ∼99% of isotype control cells. αvβ3 expression index was calculated by using the formula: mean fluorescence × percentage of αvβ3^+^ cells [4].

### MMP assay by gelatin zymography

Tumor cells were cultured for 48 h in DMEM medium containing 1% FBS in presence of pre-coated matrigel. The assay of MMP-9 in supernatants was performed as described previously [4].

### Assay of activity of NF-κB

The nuclear extract was prepared with Nuclear Extraction Kit (Millipore, Billerica, MA). The activity of NF-κB in nuclear extract was determined by NF-κB Assay kit (Millipore) according to the manufacturer's protocol.

### Cell transfection

For down-regulation of SNAI2, MCF-7 cells were transduced with SNAI2 shRNA lentiviral particles, or control shRNA lentiviral particles (Santa Cruz Biotech, Inc.) according to the manufacturer's protocol. After selection with puromycin, the cells were used for further experiments.

### Apoptosis Assay

Tumor cells were cultured for 24 h in 6-well plates pre-coated with poly-HEMA (10 mg/ml, Sigma). The cells were then stained with Annexin V-FITC/Propidium Iodide (PI) apoptosis detection kit (BD Biosciences, San Diego, CA), and analyzed by flow cytometry.

### Soft agar assay

Tumor cells were pretreated with the indicated factors for 8 days. The cells were then suspended in 0.3% agar in DMEM (20% FBS) and plated (1×10^4^ cells/well in 6-well plates) on a layer of 0.6% agar in DMEM (20% FBS) in triplicate. After 21-day culture in absence of stimuli, the cells were stained with 0.005% crystal violet. The colonies of tumor cells were counted under a microscope.

### Assay of tumor cell arrest in lung

Female athymic nude (nu/nu) mice (4 to 6 weeks old) were purchased from Beijing HFK Bio-Technology Co, LTD. (Beijing, China) for studies approved by the Committee on the Ethics of Animal Experiments of Tongji Medical College. The mice were maintained in the accredited animal facility of Tongji Medical College. The mice were randomly divided to several groups (6 mice/group). For assay of tumor cell arrest in lung, tumor cells were labeled with CFSE, and injected into mice by i.v. injection which was performed after inhalation of 2% isoflurane. 1×10^6^ cells in 0.1 ml of PBS were slowly injected after sterilization of tail with alcohol. 5 h and 24 h after the injection, the mice were sacrificed by CO_2_ inhalation. Lung tissues were harvested. Frozen sections were prepared and analyzed by fluorescence microscopy. Fluorescent spots were counted from 20 randomly chosen fields in sections of each mouse.

### Histology

Tumor cells (1×10^6^ per mouse) were injected into mice via tail vein. The lung tissues were harvested at the indicated time, and embedded in paraffin according to standard histological procedures. Tissue sections were prepared and subjected to immunohistochemical analysis or H&E staining. Immunohistochemical staining for breast cancer cell marker MGB1 (Mammaglobin A) and for proliferation marker Ki-67 was performed using anti-human MGB1 antibody and anti-human Ki-67 antibody (Abcam Biotechnology) as primary antibody. HRP-conjugated anti-rabbit IgG were used as secondary antibody. Images were obtained using OLYMPUS-BX51 microscope at 10×10, 20×10 or 40×10 magnification. Staining intensity of cells was evaluated under a microscope and graded (1, weak; 2, moderate; 3, strong) in a blinded fashion by two examiners. Staining intensity of tissue sections was assessed using a semi-quantitative immunohistochemical scoring system, HSCORE. The HSCORE was calculated using the following equation: HSCORE  = ∑Pi(i+1), where i is the staining intensity of cells and Pi is the percentage of the cells at each level of intensity [40].

### Statistical analysis

Data are pooled from three independent experiments with a total of six samples in each group. Results were expressed as mean value ± SD and interpreted by one-way ANOVA, except for the data of experimental lung metastases which were interpreted by Fisher's Exact Test. Differences were considered to be statistically significant when *P*<0.05.

## Supporting Information

Figure S1 **TGF-β1/H_2_O_2_/LPS augments polymerization of actin of tumor cells.** Tumor cells were cultured in absence or presence of TGF-β1, H_2_O_2_ and LPS for 8 days. The cells were then incubated in presence of matrigel for 5 h. The cells with highly polymerized actin were visualized by staining with rhodamine-phalloidin after incubation (left). Their percentage in total cells was calculated (right). *P* values, *P<0.05, **P<0.01.
(TIF)

Figure S2 **TGF-β1/H_2_O_2_/LPS does not significantly influence the expression of p21 and MYC.** MCF-7 cells were cultured in absence or presence of TGF-β1, H_2_O_2_ and LPS for 7 days. The expression of p21 and MYC were detected by real-time RT-PCR and Western blot.
(TIF)

Figure S3 **TGF-β1/H_2_O_2_/LPS modulates the expression of Nm23-H1 and EDG2.** MCF-7 cells were cultured in absence or presence of TGF-β1/H_2_O_2_/LPS (left) for the indicated time. Or the cells were cultured for 6 days in absence or presence of TGF-β1, H_2_O_2_, and LPS (right). The expression of *NM23-H1* **(A)**, and *EDG2* **(B)** was detected by real-time RT-PCR. *P* values, **P<0.01.
(TIF)

Figure S4 **Inhibiting PI3K suppresses TGF-β receptor expression and activation of other non-Smad pathways.** MCF-7 cells were un-treated or treated with TGF-β1/H_2_O_2_/LPS for the indicated time in absence of presence of wortmannin (WT, 40 nM). **(A)** The expression of TβRI and TβRII was detected by Western blot. The relative expression of TβRI and TβRII to β-actin was calculated after densitometric analysis of Western blot. **(B)** The phosphorylated and un-phosphorylated p38MAPK, ERK, and JNK were detected by Western blot. The ratio of phosphorylated and un-phosphorylated p38MAPK, ERK, and JNK was calculated after densitometric analysis of Western blots. The activity of NF-κB was assayed as described in Methods. *P* values, *P<0.05, **P<0.01.
(TIF)

Figure S5 **TGF-β1/H_2_O_2_/LPS promotes anoikis-resistance of non-invasive breast cancer cells.** MCF-7 and T-47D cells were cultured in absence or presence of TGF-β1/H_2_O_2_/LPS for the indicated time. The cells were then transferred to poly-HEMA-coated plate and cultured for 24 h. The apoptosis of the cells was analyzed by flow cytometry. *P* values, *P<0.05, **P<0.01.
(TIF)
