# Supplementary figures and images for: TLR4 Ligand/H2O2 Enhances TGF-β1 Signaling to Induce Metastatic Potential of Non-Invasive Breast Cancer Cells by Activating Non-Smad Pathways
Source: PLoS One. 2013 May 29;8(5):e65906. doi: 10.1371/journal.pone.0065906 (PMC3667026; doi:10.1371/journal.pone.0065906)

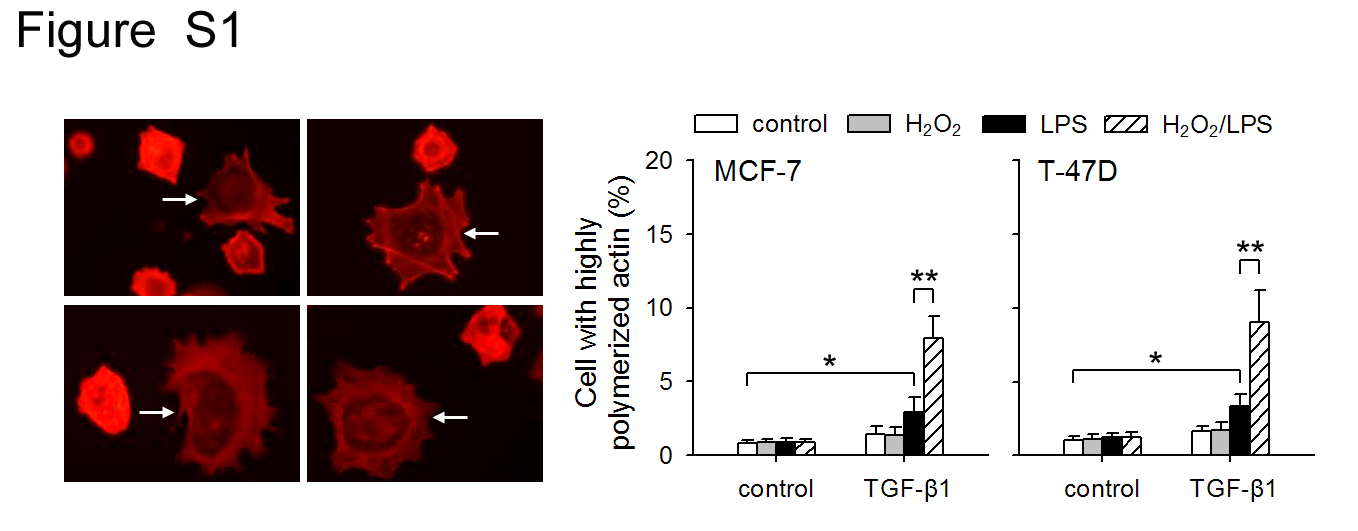

Supplement: Figure S1 — TGF-β1/H2O2/LPS augments polymerization of actin of tumor cells. Tumor cells were cultured in absence or presence of TGF-β1, H2O2 and LPS for 8 days. The cells were then incubated in presence of matrigel for 5 h. The cells with highly polymerized actin were visualized by staining with rhodamine-phalloidin after incubation (left). Their percentage in total cells was calculated (right). P values, *P<0.05, **P<0.01. (TIF) [file pone.0065906.s001.tif]

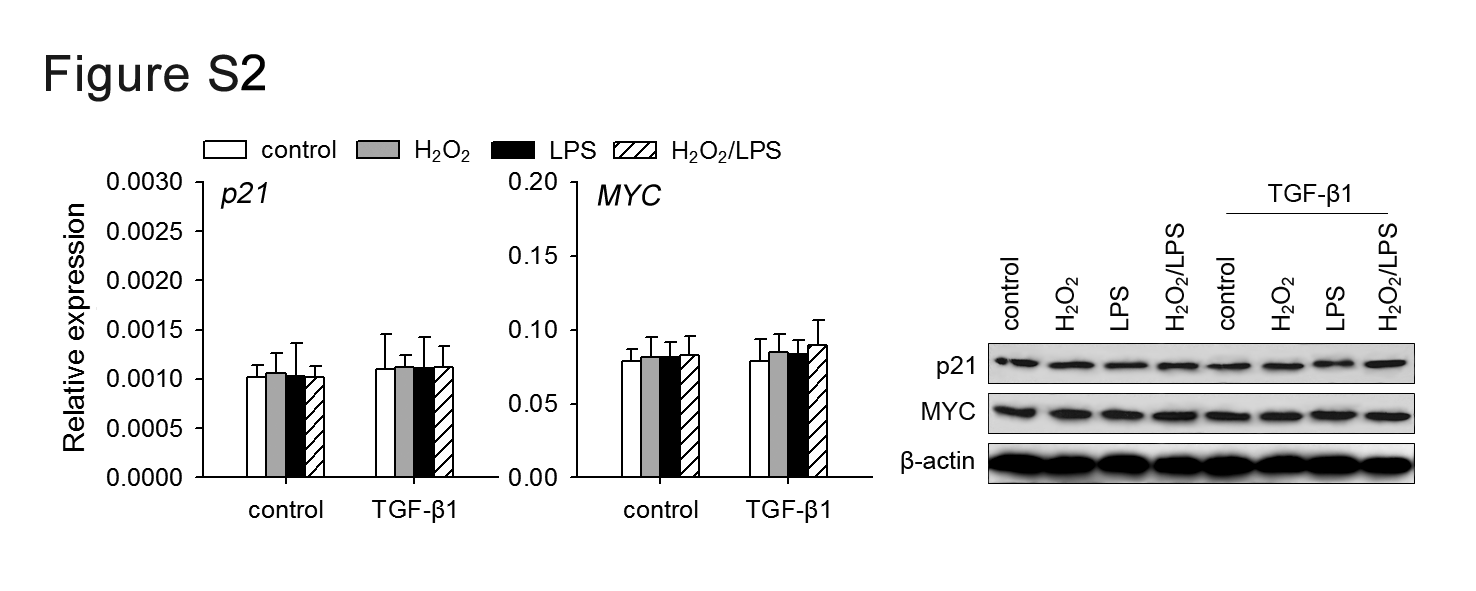

Supplement: Figure S2 — TGF-β1/H2O2/LPS does not significantly influence the expression of p21 and MYC. MCF-7 cells were cultured in absence or presence of TGF-β1, H2O2 and LPS for 7 days. The expression of p21 and MYC were detected by real-time RT-PCR and Western blot. (TIF) [file pone.0065906.s002.tif]

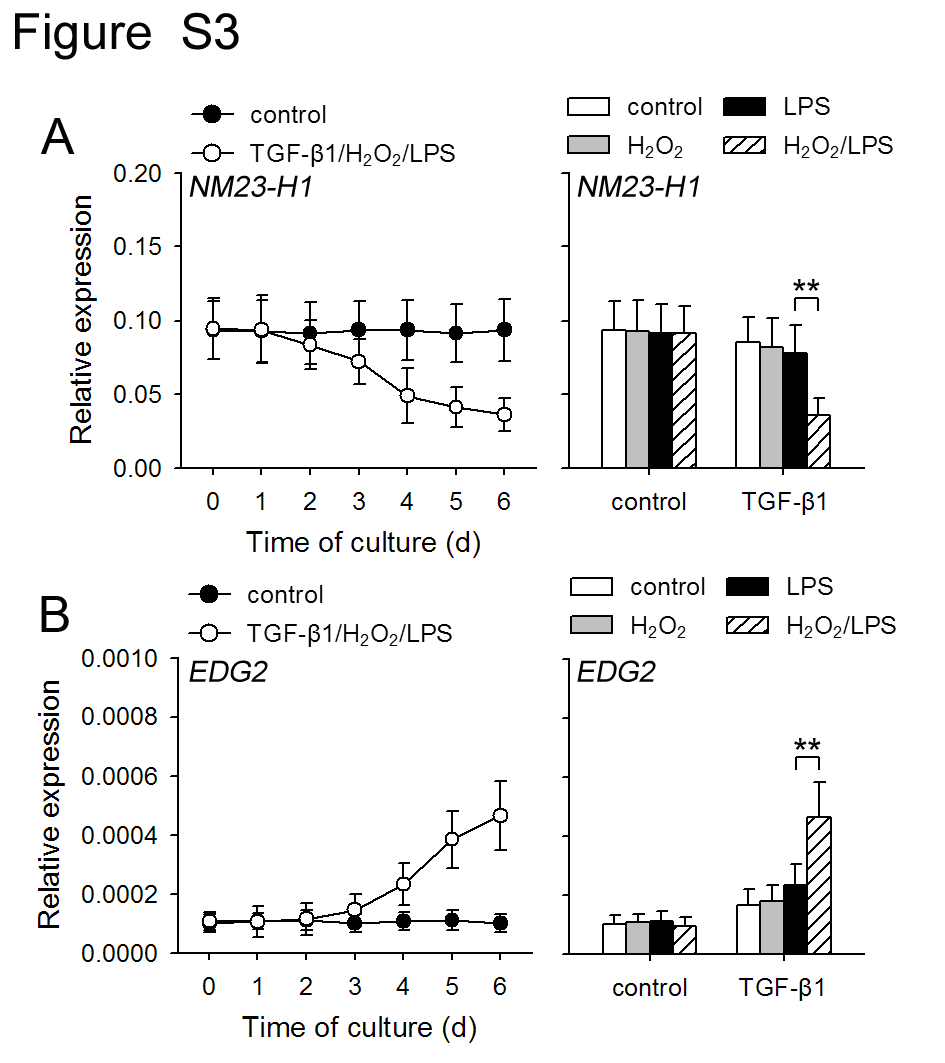

Supplement: Figure S3 — TGF-β1/H2O2/LPS modulates the expression of Nm23-H1 and EDG2. MCF-7 cells were cultured in absence or presence of TGF-β1/H2O2/LPS (left) for the indicated time. Or the cells were cultured for 6 days in absence or presence of TGF-β1, H2O2, and LPS (right). The expression of NM23-H1 (A), and EDG2 (B) was detected by real-time RT-PCR. P values, **P<0.01. (TIF) [file pone.0065906.s003.tif]

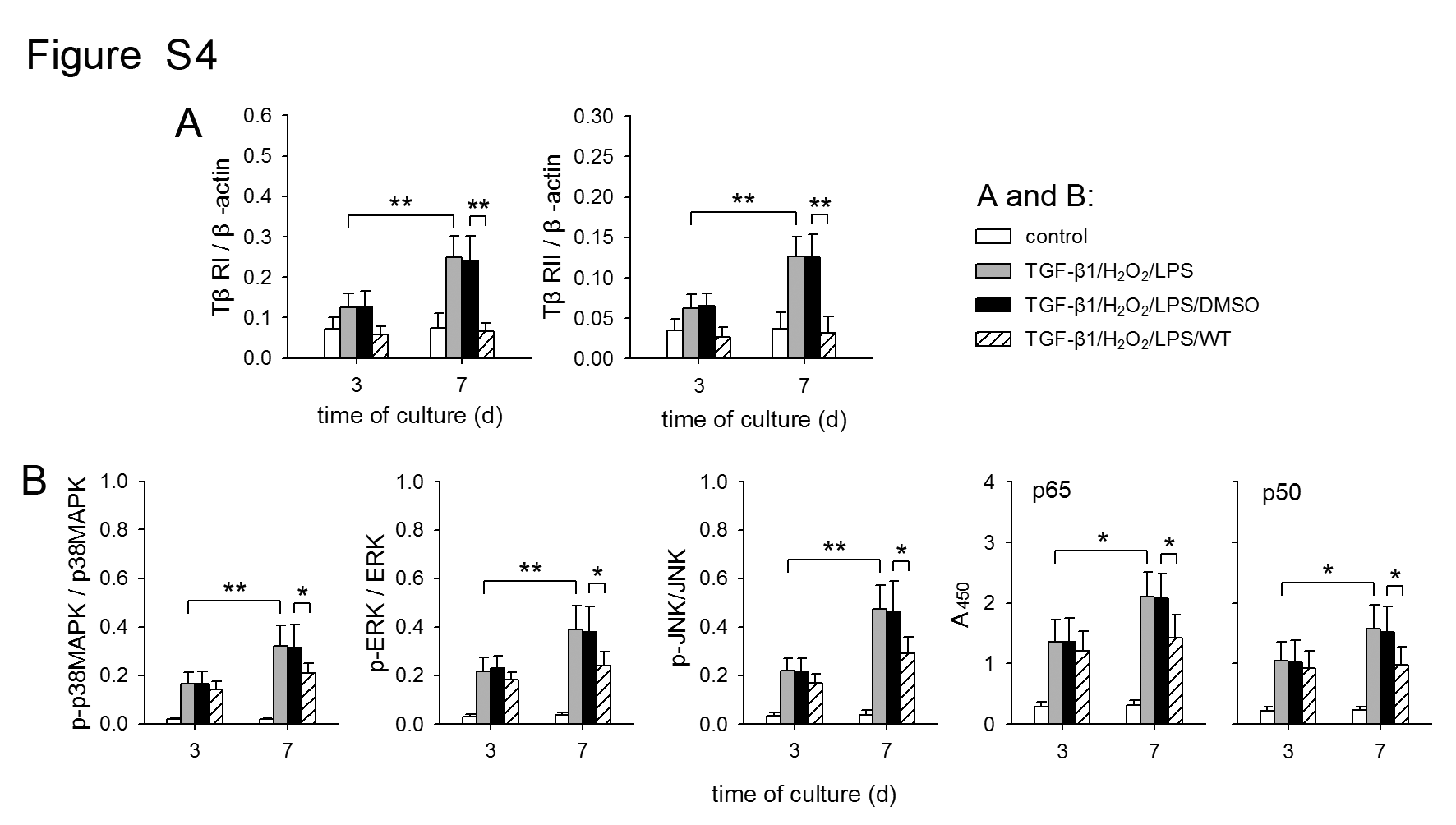

Supplement: Figure S4 — Inhibiting PI3K suppresses TGF-β receptor expression and activation of other non-Smad pathways. MCF-7 cells were un-treated or treated with TGF-β1/H2O2/LPS for the indicated time in absence of presence of wortmannin (WT, 40 nM). (A) The expression of TβRI and TβRII was detected by Western blot. The relative expression of TβRI and TβRII to β-actin was calculated after densitometric analysis of Western blot. (B) The phosphorylated and un-phosphorylated p38MAPK, ERK, and JNK were detected by Western blot. The ratio of phosphorylated and un-phosphorylated p38MAPK, ERK, and JNK was calculated after densitometric analysis of Western blots. The activity of NF-κB was assayed as described in Methods. P values, *P<0.05, **P<0.01. (TIF) [file pone.0065906.s004.tif]

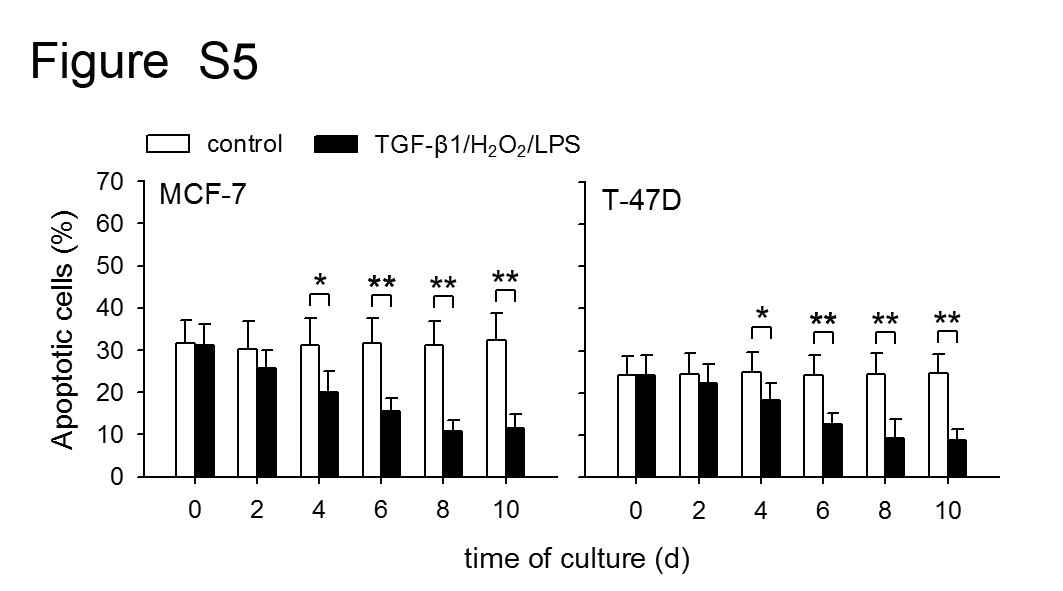

Supplement: Figure S5 — TGF-β1/H2O2/LPS promotes anoikis-resistance of non-invasive breast cancer cells. MCF-7 and T-47D cells were cultured in absence or presence of TGF-β1/H2O2/LPS for the indicated time. The cells were then transferred to poly-HEMA-coated plate and cultured for 24 h. The apoptosis of the cells was analyzed by flow cytometry. P values, *P<0.05, **P<0.01. (TIF) [file pone.0065906.s005.tif]
